# Microbial Diversity in Sediments from the Bottom of the Challenger Deep, the Mariana Trench

**DOI:** 10.1264/jsme2.ME17194

**Published:** 2018-05-25

**Authors:** Takuro Nunoura, Manabu Nishizawa, Miho Hirai, Shigeru Shimamura, Phurt Harnvoravongchai, Osamu Koide, Yuki Morono, Toshiaki Fukui, Fumio Inagaki, Junichi Miyazaki, Yoshihiro Takaki, Ken Takai

**Affiliations:** 1 Research and Development Center for Marine Biosciences, Japan Agency for Marine-Earth Science and Technology (JAMSTEC) 2–15 Natsushima-cho, Yokosuka 237–0061 Japan; 2 Department of Subsurface Geobiological Analysis and Research, Japan Agency for Marine-Earth Science and Technology (JAMSTEC) 2–15 Natsushima-cho, Yokosuka 237–0061 Japan; 3 School of Life Science and Technology, Tokyo Institute of Technology 4259 Nagatsuta, Midori-ku, Yokohama 226–8501 Japan; 4 Geomicrobiology Group, Kochi Institute for Core Sample Research, Japan Agency for Marine-Earth Science Technology (JAMSTEC) Nankoku, 783–0093 Japan; 5 Geobiotechnology Group, Research and Development Center for Submarine Resources, Japan Agency for Marine-Earth Science Technology (JAMSTEC) Nankoku, 783–0093 Japan; 6 Research and Development Center for Ocean Drilling Science, Japan Agency for Marine-Earth Science Technology (JAMSTEC) Yokohama 236–0001 Japan

**Keywords:** Hadal, Mariana Trench, nitrogen cycle, nitrification

## Abstract

The Challenger Deep is the deepest ocean on Earth. The present study investigated microbial community structures and geochemical cycles associated with the trench bottom sediments of the Challenger Deep, the Mariana Trench. The SSU rRNA gene communities found in trench bottom sediments were dominated by the bacteria *Chloroflexi* (SAR202 and other lineages), *Bacteroidetes*, *Planctomycetes*, “*Ca.* Marinimicrobia” (SAR406), and *Gemmatimonadetes* and by the archaeal α subgroup of MGI *Thaumarchaeota* and “*Ca.* Woesearchaeota” (Deep-sea Hydrothermal Vent Euryarchaeotic Group 6). The SSU rRNA gene sequencing analysis indicated that the dominant populations of the thaumarchaeal α group in hadal water and sediments were similar to each other at the species or genus level. In addition, the co-occurrence of nitrification and denitrification was revealed by the combination of pore water geochemical analyses and quantitative PCR for nitrifiers.

The hadal zone of the ocean, composed of deep trenches below a water depth of 6,000 m, is similar to the abyssal and bathyal zones in temperature, salinity, and the concentrations of oxygen, nitrate, and other major geochemical components (21, 32, 58). On the other hand, the steep and narrow geomorphology of trenches supplies the hadal zone with suspended sediments and organic compounds, possibly due to occasional landslides, and supports unique microbial ecosystems in hadal water and sediments (12, 20, 41, 68). The microbial communities in hadal water are characterized by an enrichment of potentially heterotrophic microbial populations, while those in overlying abyssal water are predominated by potentially chemolithotrophic populations (41). Microbial activity levels and cell abundance are greater in trench sediments than in adjacent abyssal plain sediments, and the trench benthic microbial ecosystem maintains the efficient mineralization of organic compounds (12, 68).

The Mariana arc-trench system is a non-accretionary convergent plate margin between the Philippine Sea plate and subducting Pacific plate (44). In contrast to the other trenches located in the North-West Pacific region, the Mariana Trench is topographically isolated from other trenches by the impinging Ogasawara Plateau and Caroline Ridge (19). Thus, these topographical barriers may interrupt the transfer of organic compounds and (micro-) organisms entrained by inter trench currents. Microbiological studies on hadal benthic habitats started in the 1950s based on culture-dependent techniques (1, 21), and bottom sediments in the Mariana Trench are one of the most intensively studied environments in the history of hadal benthic microbiology (22, 23, 25, 27, 30, 33, 46–48, 59, 61–64, 66, 71). However, microbial diversity and functions related to the benthic geochemical cycle in the hadal sediment have not yet been examined in detail, although greater microbial activity and abundance have been reported in hadal sediments than in adjacent abyssal plain sediments (12). In the present study, we investigated microbial community structures and the nitrogen cycle as well as geochemical gradients in hadal sediments of the Challenger Deep, the Mariana Trench.

## Materials and Methods

### Site description and sampling

Three dives of the ROV ‘*ABISMO*’ were conducted in the Challenger Deep of the Mariana Trench (11°22.25′N, 142°42.75′E, 10,300 m) during the JAMSTEC *R/V Kairei* KR08-05 cruise (June 2008) (70). Temperature, depth, and salinity were measured using an SBE49 CTD sensor (Sea-Bird Electronics, Bellevue, WA, USA). Water sampling from the sea surface to near the bottom of the trench (water depth of 10,257 m) and sample processing and storage were described previously (41). A sediment core (#AB11) was obtained from the trench bottom by a gravity corer on the ROV ‘*ABISMO*’ in the same dive. Sediment cores were divided by every 5 cm above 20 cm below the seafloor (cmbsf) and every 10 cm below 20 cmbsf, and subsamples for molecular analyses were stored at −80°C. Pore water from each section was obtained by centrifugation at 4,000×*g* for 10 min and filtration using a syringe cartridge filter (0.2 mm). Pore water samples for nutrient analyses were stored at −20°C.

### Geochemical analyses

Pore water pH and alkalinity were measured onboard using a 794 Basic Titrino titration system (Metrohm, Herisau, Switzerland), and potentiometric titration was performed with 0.1 M HCl per 2 mL of pore water. The pore water samples used for SO_4_^2−^ and nutrient concentration measurements were filtrated using a cartridge filter with a pore size of 0.2 μm. SO_4_^2−^ concentrations were analyzed using the personal ion analyzer PIA-1000 (Shimadzu, Kyoto, Japan). The remaining pore water samples were stored at −20°C. After the cruise, NO_3_^−^, NO_2_^−^, PO_4_, and NH_4_^+^ were analyzed spectrophotometrically using an automated QuAAtro 2-HR analyzer (BL TEC, Osaka, Japan).

The contents and stable isotopic ratio of total organic carbon and nitrogen were estimated with a Flash EA1112 elemental analyzer (Thermo Fisher Scientific, Waltham, MA, USA) at SI Science (Sugito-machi, Saitama, Japan).

The stable nitrogen and oxygen isotopic compositions of pore water nitrate were assessed using the denitrifier method (6, 55). After the removal of nitrite by the addition of sulfamic acid to pore water samples (14), nitrate was quantitatively converted to nitrous oxide using the strain *Pseudomonas chlororaphis* (JCM20509= ATCC13985), a denitrifying bacterium lacking the ability to reduce N_2_O. After the online extraction and purification of the resultant nitrous oxide (36), nitrogen and oxygen isotopic ratios were measured using a continuous-flow isotope ratio mass spectrometer (CF-IRMS) (Delta V Plus, Thermo Fisher Scientific) at JAMSTEC. Corrections for the drift, size, and fractionation of oxygen and nitrogen isotopes during the procedures were made using international nitrate standards: USGS32, USGS34, USGS35, and IAEA-NO_3_.

The isotope ratios of ^13^C/^12^C, ^15^N/^14^N, and ^18^O/^16^O are reported as δ values, in which δ=(R_sam_/R_std_–1)×1,000, with R being the isotope ratios in the sample and standard, respectively. The carbon isotope ratio of total organic carbon was referenced against Vienna Pee Dee Belemnite (V-PDB). The nitrogen isotope ratios of total nitrogen and nitrate were referenced against atmospheric N_2_, and the δ^18^O values of nitrate were assessed relative to Vienna Standard Mean Ocean Water (V-SMOW).

The precision rates achieved through repeated analyses of an in-house nitrate standard were typically better than 0.2‰ for δ^15^N and δ^18^O.

### Cell abundance

The abundance of microbial cells in the sediment samples was measured using an image-based, discriminative cell-counting technique described previously (34). In brief, a paraformaldehyde-fixed slurry sample was mildly sonicated at 5 W for 1 min with an UH-50 ultrasonic homogenizer (SMT, Tokyo), and then treated with 1% hydrogen fluoride (HF) for 20 min. An aliquot was filtered onto polycarbonate filters with a pore size of 0.2 μm (Isopore; Merck Millipore, Billerica, MA, USA). A microscopic count of SYBR Green I-stained cells was performed by an automated SYBR DiCE system (34), and >140 of acquired images were processed by MetaMorph software (Molecular Devices, Downingtown, PA, USA).

### DNA extraction

Approximately 5 mL of each of the frozen sediment samples from certain sections of the sediment core was used for DNA extraction. Environmental DNA was extracted using a PowerMax Soil DNA Isolation Kit (MoBio Lab, Carlsbad, CA, USA) with minor modifications.

### Sequencing analyses

The amplification conditions and primer sequences for each PCR amplification are summarized in Table S1. In clone analyses of archaeal and bacterial SSU rRNA genes, the primer sets Arch21F/Arch958R (8) and Bac27F/Bac927R (8, 28) were used, respectively, for PCR amplification. In the tag sequencing of the prokaryotic SSU rRNA gene, the primer set 530F/907R with 10 bp of extended tag sequences in the 5′ termini was used for SSU rRNA gene amplification (39). These SSU rRNA gene fragments were amplified from environmental DNA assemblages using LA *Taq* polymerase with GC buffer (Takara Bio, Kusatsu, Japan).

In the archaeal *amoA* clone analysis, the primer set arch-amoAF/arch-amoAR (10) was used with EX *Taq* polymerase (Takara Bio) to amplify gene fragments from the environmental DNA assemblages as described previously (40). In order to amplify *hzsA*, the primer set hzsA 526F/hzsA 1857R (17) was applied using LA *Taq* DNA polymerase with Mg^2+^ buffer as described previously (40). In the amplification of *mcrA*, the primer set ME3MF/ME2r’ (38) was used with SYBR Premix Ex *Taq* II (Takara Bio) in the presence of MgCl_2_ (final 2.5 mM) as described previously (38).

In clone analyses, amplified DNA fragments were cloned into the pCR2.1 vector (Invitrogen), and clone libraries were constructed. The inserts were directly sequenced with the M13M4 primer using an ABI3730xl genetic analyzer with Big Dye v. 3.1 (Applied Biosystems, Foster City, CA, USA). SSU rRNA gene sequences with >97% identity were assigned the same clone type (phylotype) based on FastGroup II (72) and a similarity analysis in GENETYX-MAC v. 15 (GENETYX, Tokyo, Japan). In the clone analysis, representative SSU rRNA gene sequences were aligned and phylogenetically classified into certain taxonomic units using ARB (50). Archaeal *amoA* gene sequences with >96% identity were assigned the same phylotype, and the phylogenetic tree was constructed by Clustal X based on unambiguous residues (29).

The SSU rRNA gene amplicons for tag sequencing were analyzed by a 454 FLX Titanium sequencer (Roche, Basel, Switzerland). All of the raw tag sequences were analyzed with the shhh.flows pipeline in MOTHUR 1.31.1 (51, 53, 54), and the primer sequences in either or both ends of the tags were eliminated. Tag sequences shorter than 300 bp were removed from downstream analyses. Potentially chimeric sequences were surveyed using UCHIME (9). Phylogenetic assignments and statistical analyses for the tag sequences were then conducted. Sequencing tags were aligned using the partial order algorithm (POA) (SINA, http://www.arb-silva.de/aligner/) with SILVA SSU Ref NR as a reference for multiple alignments (69). All of the aligned sequences were then clustered into operational taxonomic units (OTUs) based on 97% sequence identity using MOTHUR 1.31.1 with the default parameters according to the average-clustering algorithm. The output was then parsed to produce occurrence tables of each OTU in each sample. The taxonomic position of each OTU was automatically assigned based on a BLAST analysis in the QIIME software package (5) using SILVA Ref NR as a reference dataset of SSU rRNA gene sequences. The sequences that are closely related to the potential contaminants belonging to genera that inhabit soil and the human body and that have been detected from negative control experiments of environmental microbiology in our lab, such as *Bradyrhizobium*, *Brevundimonas*, *Burkholderiaceae*, *Delftia*, *Erythrobacter*, *Lactococcus*, *Legionella*, *Methylobacterium*, *Mycobacterium*, *Neisseria*, *Novosphingobium*, *Propionibacterium*, *Sphingobium*, *Sphingomonas*, *Sphingopyxis*, *Staphylococcus*, *Stenotrophomonas*, and *Streptococcus*, were excluded (43). Sequences with relatively high E-values (>1.0E–30) or low identity (<90%) to the best match sequence were designated as other archaea or bacteria, and sequences that did not show significant similarity to any reference sequences were also excluded from the analysis. Alpha diversity indices (rarefaction curves, Chao1, ACE, Shannon, Shannon evenness, and Simpson) in each library and taxa/divisions were also calculated using MOTHUR 3.6. Jaccard and Bray-Curtis dissimilarity indices were estimated for each library using the vegan package in the R environment (http://vegan.r-forge.r-project.org). Phylogenetic trees were constructed from the curated alignment of representative sequences using the Clustal W program (http://www.clustal.org). A weighted UniFrac distance matrix of sediment samples was constructed from the phylogenetic tree and a sample mapping files that showed the frequencies of the sequence tags within OTUs. Sequencing tags for each phylum/class were collected from the entire dataset in accordance with the taxonomic position of each OTU. Sub-datasets were analyzed in the same manner as the entire dataset.

### Quantitative PCR (qPCR) analyses

The primers, probes, components of the standard mixture, and amplification conditions used for each qPCR are summarized in Table S1. The abundance of each gene was quantified as an average of duplicate or triplicate analyses. The 7500 Real Time PCR System (Applied Biosystems) was used for all qPCR analyses.

The quantification of prokaryotic and archaeal SSU rRNA genes was conducted using the primer and probe sets Uni340F/Uni516F/Uni806R and Arch349F/Arch516F/Arch806R, respectively (60). The abundances of *Nitrospina*, Seafloor *Nitrospina*-Like group (SFLNG) and *Nitrospira* SSU rRNA genes were also examined according to methods described previously (13, 40). In order to quantify archaeal *amoA*, group-specific primer sets for groups A, Ba, Bb, and D (2, 41) were used for qPCR. Betaproteobacterial *amoA* was also quantified as described previously (57). Amplified products from qPCR using the SYBR Premix reagent were confirmed by agarose gel electrophoresis. In order to prepare qPCR mixtures, qPCR Quick GoldStar Mastermix Plus (Eurogentec, Seraing, Belgium) was applied for the SSU rRNA genes of archaea, all prokaryotes, and *Nitrospira*, and SYBR Premix Ex Taq II (Takara Bio) was used for archaeal and betaproteobacterial *amoA* genes and *Nitrospina* and Seafloor *Nitrospina*-Like group (SFNLG) SSU rRNA genes.

### Accession numbers

The nucleotide sequences obtained in this study have been deposited in the DDBJ/EMBL/GenBank database: environmental SSU rRNA and archaeal *amoA* gene sequences (LC049988–LC050107 and LC050108–LC050123, respectively), and in the Short Read Archive database: environmental SSU rRNA tags (DRA006342).

## Results and Discussion

### Pore water chemistry and organic geochemistry

**Fig. 1:**
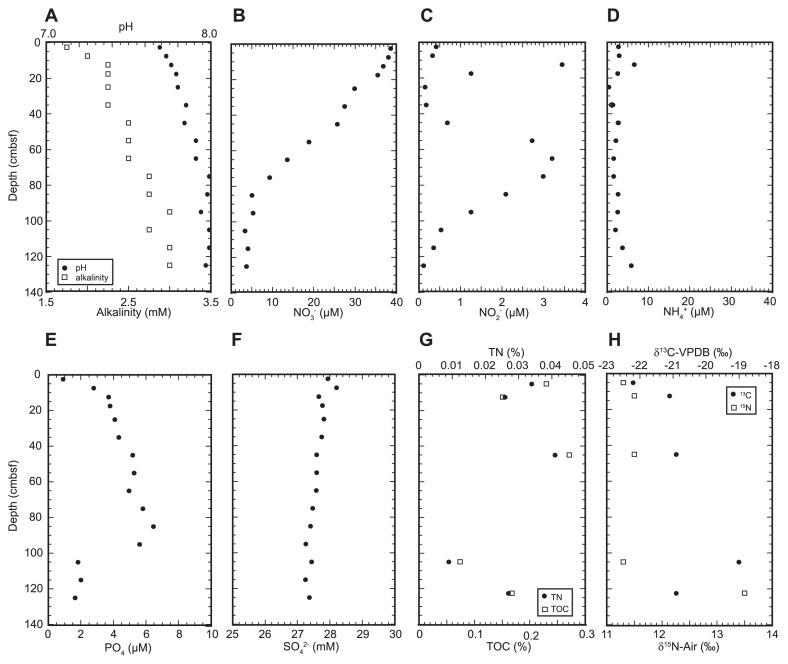
Pore water geochemistry: alkalinity and pH (A), and concentrations of NO_3_^−^ (B), NO_2_^−^ (C), NH_4_^+^ (D), PO_4_ (E), and SO_4_^2−^ (F). Sediment organic geochemistry: total organic compounds (TOC) and total nitrogen (TN) (G), as well as δ^13^C TOC and δ^15^N TN (H) of sediment core #AB11 taken from the Challenger Deep, the Mariana Trench.

The nutrient (NH_4_^+^, NO_3_^−^, NO_2_^−^, and PO_4_) and SO_4_^2−^ concentrations, pH, and alkalinity of pore water were analyzed for the sediment core #AB11, which was taken from the Challenger Deep (water depth of 10,300 m) (Fig. 1 and Table S2). NO_3_^−^ concentrations were depleted to less than approximately 5 μM below 85 cmbsf. The NO_2_^−^ profile presented two peaks: a shallow peak at 15 cmbsf and a deeper peak between 45 and 105 cmbsf. The PO_4_ profile presented an anomalous inflection at 1 mbsf. The SO_4_^2−^ profiles indicated that microbial sulfate reduction was negligible in the sediment core.

Total organic carbon (TOC) and total nitrogen (TN) ranged between 0.074 and 0.27 wt% and between 0.009 and 0.041 wt%, respectively. The stable isotopic ratios of the organic compounds δ^13^C and δ^15^N ranged between −19.0 and −22.2‰ and between 11.3 and 13.5‰, respectively (Fig. 1). The TOC concentrations obtained in the present study were slightly lower than those reported previously for Challenger Deep sediments (12). δ^15^N values at this site were close to the highest δ^15^N value of sedimentary organic compounds in the modern ocean (65), and may have originated from primary production with highly ^15^N-enriched nitrate transported on the North Equatorial current.

### Interpretation of δ^15^N_NO3−_ and δ^18^O_NO3−_

**Fig. 2:**
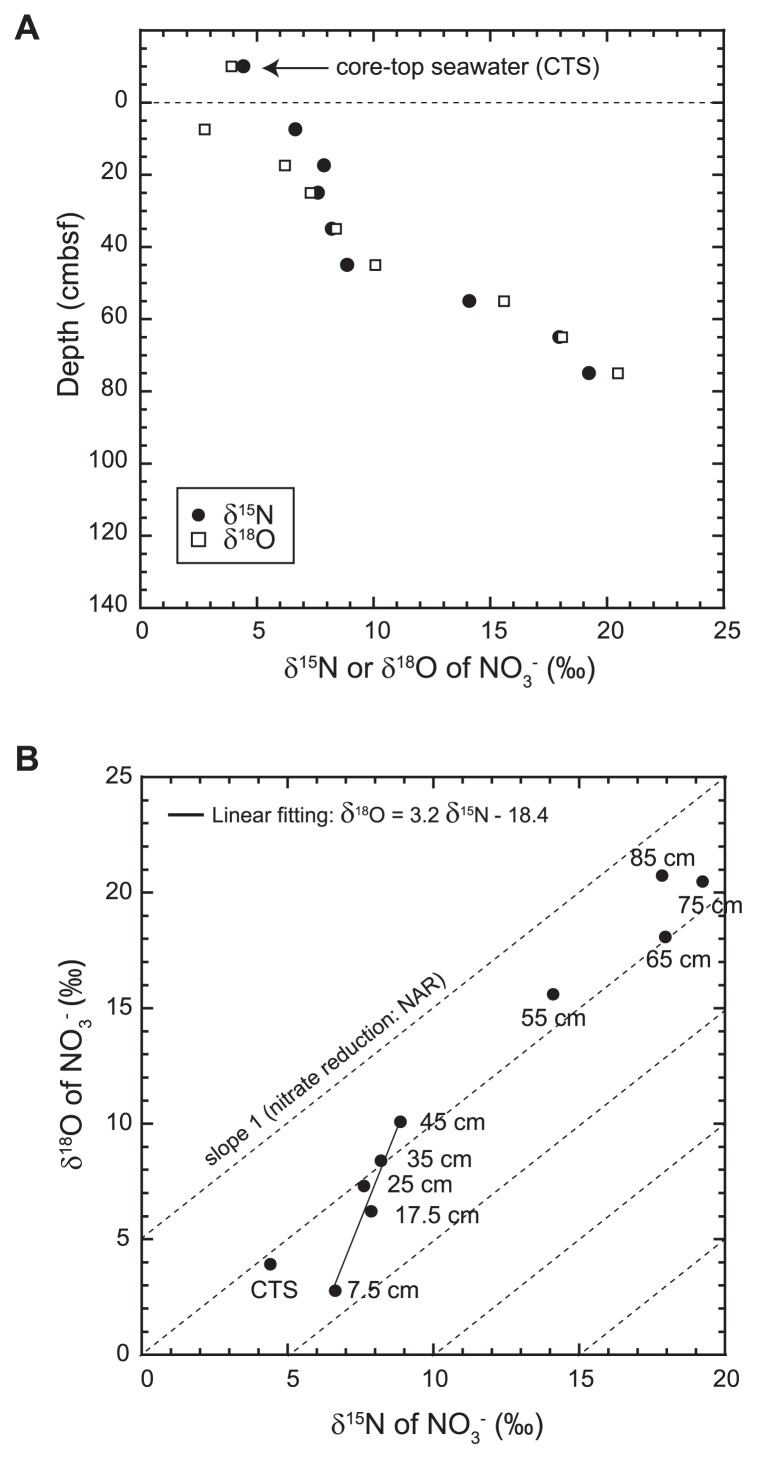
(A) δ^15^N and δ^18^O profiles of pore water NO_3_^−^ in the sediment core #AB11 taken from the Challenger Deep, the Mariana Trench. (B) The relationship between pore water nitrate δ^15^N and δ^18^O values. In comparisons, based on experimental results, fractionation lines constraining changes in nitrate δ^15^N and δ^18^O values during nitrate reduction were plotted (slopes ^18^ɛ/^15^ɛ=1) (14, 16).

The δ^15^N value of nitrate in pore water (δ^15^N_NO3−_) ranged between 4.4 and 17.8‰, and the δ^18^O_NO3−_ value ranged between 2.8 and 20.7‰. δ^15^N_NO3−_ and δ^18^O_NO3−_ values increased with depth up to 75 cmbsf (Fig. 2A and Table S2). The δ^15^N–δ^18^O relationship of pore water nitrate revealed signatures to estimate the potential contributions of the *in situ* nitrate supply (nitrification) and consumption (dissimilatory nitrate reduction) to the dynamic nitrogen cycle in the subsurface biosphere (40, 67). The δ^15^N–δ^18^O relationship of pore water nitrate changed at a depth of 50 cmbsf. The δ^15^N_NO3−_–δ^18^O_NO3−_ relationship exhibited a slope of 3.2 (±0.5) in the shallower interval above 50 cmbsf, and 0.95 (±0.10) in the deeper interval between 50 cmbsf and 80 cmbsf. The down-core decrease in the slope of the δ^15^N_NO3−_–δ^18^O_NO3−_ relationship was also observed for trench bottom sediments in the Ogasawara Trench (40).

The similar increases observed in δ^15^N_NO3−_ and δ^18^O_NO3−_ values with decreases in nitrate concentrations in the deeper interval may be explained by equivalent nitrogen vs oxygen isotope fractionation during denitrification (*i.e.*, the preferential breakage of ^14^N-^16^O bonds by respiratory nitrate reductase) (15) (Fig. 2B). In contrast, a preferential increase in δ^18^O_NO3−_ over δ^15^N_NO3−_ with decreases in nitrate concentrations in the shallower interval required the involvement of nitrification because the δ^15^N and δ^18^O values of nitrate from nitrification (δ^15^N_NTR_ and δ^18^O_NTR_) are set independently of each other. In the shallower interval, ammonium from the remineralization of organic matter and nitrite did not accumulate with depth, except for 12.5 cmbsf and 17.5 cmbsf, as discussed below. Thus, δ^15^N_NTR_ was expected to be closer to the δ^15^N of ambient organic matter (11‰; Fig. 1H) because the δ^15^N of ammonium is generally similar to that of host organic matter (±1‰) (11, 49). In contrast, δ^18^O_NTR_ is mainly set by the δ^18^O values of seawater (0‰) and dissolved O_2_ (≥23.5‰) as well as kinetic isotope effects during the multistep oxidation of ammonium to nitrite (3, 7, 37). Using available data on oxygen isotope effects during nitrification, the δ^18^O_NTR_ value in oceanic sediments was estimated to have a value between −6 and 0‰ when the δ^18^O value of pore water O_2_ (δ^18^O_O2_) was equal to that of bottom seawater (*ca.* 27‰). The calculation of δ^18^O_NTR_ was shown below. Furthermore, the δ^18^O_NTR_ value in oceanic sediments is expected to increase to ≥5‰ with depth due to downward increases in δ^18^O_O2_ by aerobic respiration (^18^ɛ=20±3‰) (24). For example, the δ^18^O_NTR_ value was predicted to increase to 6~11‰ when 88% of dissolved O_2_ in bottom seawater was consumed in sediments (δ^18^O_O2_=70‰). Therefore, the depth profile of dual isotope compositions and nitrate concentrations in pore water may be mostly explained by the coupling of nitrification and denitrification in the shallower interval, whereas denitrification in the deeper interval though the depth profile of δ^18^O_O2_ was not measured at this site.

A metabolic shift from the coupling of nitrification and denitrification to denitrification with depth is consistent with the nutrient concentration profiles in pore water. In the shallower interval, phosphate concentrations and alkalinity synchronously increased with depth, while ammonia concentrations did not change significantly (Fig. 1). Nitrate concentrations decreased with depth. These contrasting changes suggest nitrification coupled with an ammonia supply from the remineralization of organic matter via aerobic and anaerobic (=nitrate) respiration in the shallower interval. In contrast, in the deeper interval, phosphate and ammonium concentrations and alkalinity did not change significantly with depth, whereas nitrate concentrations decreased with depth. These changes do not support nitrification, but suggest denitrification.

### Calculation of the δ^18^O_NTR_ value

The δ^18^O value of nitrate produced from the sequential oxidation of ammonia via nitrite (δ^18^O_NTR_) is expressed as follows when the intermediate nitrite pool does not accumulate (4): δ^18^O_NTR_=(1–X_NOB_) (2/3*δ^18^O_NO2−,pro_+1/3*[δ^18^O_H2O_–^18^ɛ_k,H2O,NOB_]) +(δ^18^O_H2O_+^18^ɛ_eq_)(X_NOB_) (eq. 1), where X_NOB_ is the fraction of nitrite oxygen atoms that have equilibrated with water catalyzed by NOB. δ^18^O_NO2−,pro_ is the δ^18^O value of nitrite produced from archaeal and bacterial ammonia oxidation. The kinetic isotope effect for oxygen atom incorporation from water is denoted as ^18^ɛ_k,H2O,NOB_, whereas the equilibrium fractionation of oxygen isotopes between nitrite and water is denoted as ^18^ɛ_eq_ (15‰ at 4°C).

Previous culture experiments showed that ambient water and dissolved oxygen are oxygen sources for nitrite produced during archaeal and bacterial ammonia oxidation (7, 37). In their experiments, the δ^18^O value of nitrite produced from archaeal and bacterial ammonia oxidation (δ^18^O_NO2−,pro_) was expressed as follows: δ^18^O_NO2−,pro_={0.5(δ^18^O_O2_–^18^ɛ_k,O2,AO_)+ 0.5(δ^18^O_H2O_–^18^ɛ_k,H2O,AO_)}(1–X_AO_)+(δ^18^O_H2O_+^18^ɛ_eq_)(X_AO_)= 0.5(1+X_AO_)δ^18^O_H2O_+0.5(1–X_AO_)(δ^18^O_O2_–^18^ɛ_k,O2,AO_–^18^ɛ_k,H2O,AO_) +(X_AO_)^18^ɛ_eq_ (eq. 2), where XAO is the fraction of nitrite oxygen atoms that have equilibrated with water catalyzed by AOA or AOB. The kinetic isotope effects for oxygen atom incorporation from dioxygen and water are denoted as ^18^ɛ_k,O2,AO_ and ^18^ɛ_k,H2O,AO_, respectively.

Using experimental data on X_AO_, ^18^ɛ_k,O2,AO_+^18^ɛ_k,H2O,AO_, and ^18^ɛ_eq_ (37), the potential δ^18^O_NO2−,pro_ value in trench bottom sediments was estimated (−1‰ to 7‰ if δ^18^O_O2_=27‰). The potential δ^18^O_NTR_ value in trench bottom sediments was then estimated from equation 1 using the experimental values of X_NOB_ (0.02) and ^18^ɛ_k,H2O,NOB_ (15‰) (3). We assumed 27‰ as the δ^18^O_O2_ value of bottom seawater in the Mariana Trench (170 μmol O_2_ kg^−1^) based on the empirical relationship between δ^18^O_O2_ and O_2_ concentrations in the deep ocean (26). The experimental value of ^18^ɛ_eq_ at 4°C was previously reported to be 15‰ (4).

### Microbial abundance

**Fig. 3:**
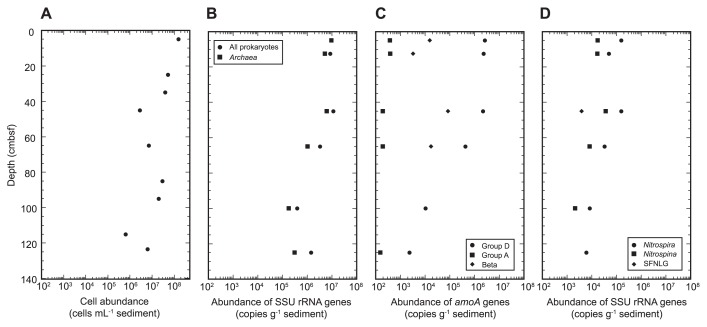
Profiles of direct cell counts (A) and copy numbers of whole prokaryotic and archaeal SSU rRNA genes (B), *amoA* genes (C), and SSU rRNA genes of nitrite oxidizers (D) in sediment core #AB11 taken from the Challenger Deep, the Mariana Trench. Groups D and A and Beta (C) indicate the archaeal *amoA* of groups D and A and betaproteobacterial *amoA*, respectively. SFNLG (D) indicates the potential nitrite-oxidizing Subseafloor *Nitrospina*-Like Group.

Cell abundance above 123 cmbsf ranged between 6.3×10^5^ and 1.5×10^8^ cells mL^−1^ sediment (Fig. 3). In the qPCR analysis, the abundance of the entire prokaryotic SSU rRNA gene ranged between 3.9×10^5^ and 1.1×10^7^ copies g^−1^ sediment. The copy number of the archaeal SSU rRNA gene ranged between 1.8×10^5^ and 9.4×10^6^ copies g^−1^ sediment. These results showed that microbial abundance generally decreased with increases in depth with a few exceptions; however, several gaps were observed between direct cell counts and SSU rRNA gene copy numbers. These gaps may have been the result of biases in DNA extraction, PCR inhibitors in environmental DNA solutions, primer and probe mismatches, and variance in SSU rRNA copy numbers, as discussed previously (*e.g.*, 18, 31, 35). The relative abundance of the archaeal SSU rRNA gene in all of the prokaryotic SSU rRNA genes estimated by qPCR was higher in surface sediments and lower in core bottom sediments.

### SSU rRNA gene diversity analyses

SSU rRNA gene diversity was analyzed using cloning and tag-sequencing techniques. Samples at depths of 5, 12.5, 45, 65, 100, and 123 cmbsf were used for the SSU rRNA gene clone analysis, and those at depths of 5, 12.5, 65, and 123 cmbsf were used for SSU rRNA gene tag sequencing.

A total of 267 bacterial SSU rRNA gene sequences (42 to 47 sequences for each sample) and 322 archaeal SSU rRNA gene sequences (44 to 90 sequences for each sample) were obtained in the clone analysis (Table S3 and S4). Bacterial SSU rRNA gene communities were generally similar to each other, except for the sample from a depth of 123 cmbsf. Sequences from *Alphaproteobacteria*, *Chloroflexi*, and *Gemmatimonadetes* were detected as the dominant populations in all of the bacterial clone libraries, and the relative abundance of *Chloroflexi* phylotypes increased in the sample from a depth of 123 cmbsf. The SAR202 group was abundant in the *Chloroflexi* population in the libraries from depths above 100 cmbsf; however, its relative abundance decreased at a depth of 123 cmbsf. Archaeal SSU rRNA gene clone libraries were dominated by only one phylotype MCD_AB10_5cm_A01, which belonged to the α subgroup of the Marine Group I (MGI) *Thaumarchaeota*, including the genus *Nitrosopumilus*. MCD_AB10_5cm_A01 was very similar to the hadal water phylotype MW_10000m_04 (similarity >98%). Subgroup γ MGI, another abundant MGI population in hadal water (41), did not dominate in benthic archaeal 16S rRNA gene communities.

**Fig. 4:**
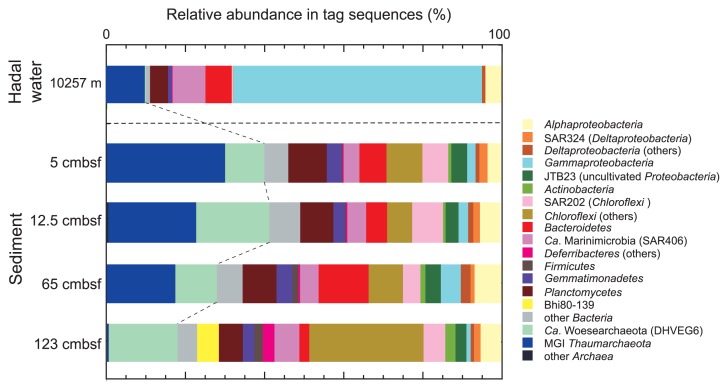
Composition of SSU rRNA gene tags of microbial communities from hadal water and trench bottom sediment (sediment core #AB11) in the Challenger Deep, the Mariana Trench.

As a result of tag sequencing, 53886 rRNA gene tags (each from 10547 to 12402 tags) were obtained from the four DNA assemblages after removing non-SSU rRNA gene tags, shorter tags, potential contaminants, and chimeric sequences (Fig. 4 and Table S5). SSU rRNA gene tag populations were generally similar to the SSU rRNA gene communities shown by the clone analysis. However, several differences were found between the SSU rRNA gene communities obtained by the two techniques. A notable difference was that “*Candidatus* (Ca.) Woesearchaeota” (Deep-sea Hydrothermal Vent Euryarchaeotic Group 6 [DHVE6]) (45) was dominant in the tag communities, but was not detected in the classical clone analysis. Moreover, the abundance of the MGI tags markedly decreased at a depth of 123 cmbsf, whereas the MGI population dominated in all of the archaeal SSU rRNA gene clone libraries; the relative abundance of MGI tags in all of the archaeal tags in a sample from a depth of 65 cmbsf was 62%, and that from a depth of 123 cmbsf was 2%. The relative abundances of *Bacteroidetes* and *Planctomycetes* in the tag communities were also higher than those in the SSU rRNA gene libraries. At several depths, a potentially methanogenic/methanotrophic archaeal lineage was found to be a minor population in the tag community and clone library.

### Amplification and diversity of functional genes; *amoA*, *mcrA*, and *hzsA*, and qPCR of *amoA* genes

The amplification of the archaeal *amoA* and *mcrA* genes and *hzsA* was examined in order to detect the presence of archaeal ammonia oxidizers, methanogens/anaerobic methanotrophs, and anaerobic ammonium oxidizers. As a result, only the amplicons of archaeal *amoA* genes were obtained.

The archaeal *amoA* gene communities, which consisted of five subgroups, were more diverse than those in the thaumarchaeal communities observed in the SSU rRNA gene clone analysis (Table S6). The grouping of archaeal *amoA* in the present study was based on previous studies (2, 10, 41). The most abundant phylogroup was the Ba subgroup, with subgroups A and D as predominant populations. Coordination between the thaumarchaeal SSU rRNA and *amoA* gene clusters was suggested as follows: group α in the SSU rRNA gene and D in *amoA*, β and A, γ and Ba, and δ and Ba (41). Thus, the predominance of group D was expected in the archaeal *amoA* gene communities based on the SSU rRNA gene clone analysis, while it was not the most abundant population in the *amoA* clone analysis. A similar contradiction between the SSU rRNA and *amoA* gene clone analyses was also observed in a previous water column study on the Mariana Trench, and the contradiction was suggested to be a result of biases in the *amoA* gene clone analysis (41).

We then investigated the abundance of each group of archaeal *amoA* genes using group-specific primer sets. As a result, only subgroups A and D were detected in qPCR, while Ba and Bb were not (Fig. 3). The maximum abundance of subgroup D *amoA* (2.6×10^6^ copies g^−1^ sediment) was found in the surface layer and decreased with increasing depth. The abundance of subgroup A *amoA* was less than 3.8×10^2^ copies g^−1^ sediment. Considering the detection limits and experimental system of qPCR for subgroups Ba and Bb, the abundances of these groups in the surface sediments were less than 2.9×10^4^ and 5.7×10^2^ copies g^−1^ sediment, respectively. This result is consistent with the results of the SSU rRNA gene community analyses that indicated the predominance of group α MGI in the thaumarchaeal populations. Betaproteobacterial *amoA* was also detected in the sediments, with maximum abundance being found at 45 cmbsf (8.4×10^4^ copies g^−1^ sediment).

### qPCR analyses for SSU rRNA of nitrite oxidizers

The abundance of nitrite oxidizers was examined using group-specific SSU rRNA gene qPCR to target *Nitrospira*, *Nitrospina*, and a potential nitrite-oxidizing SFNLG (40) (Fig. 3). The results obtained indicated that *Nitrospira* members were the most abundant nitrite oxidizer, and its abundance peaks (1.5 and 1.6×10^5^ copies g^−1^ sediment) were found in samples from 5 and 45 cmbsf, respectively. The second most abundant nitrite oxidizer was shown to be *Nitrospina* members, with maximum abundance (3.7×10^4^ copies g^−1^ sediment) being detected in a sample from 45 cmbsf. A potential nitrite-oxidizing SFNLG was a minor population.

### Gaps among PCR-dependent analyses for archaeal communities

**Fig. 5:**
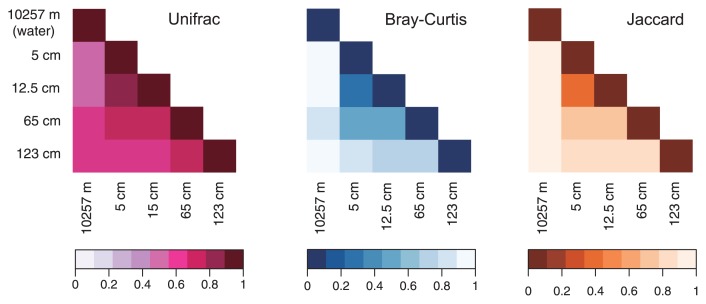
Matrices of UniFrac distances and Bray-Curtis and Jaccard dissimilarity indices of SSU rRNA gene communities obtained by tag sequencing for trench bottom sediments in the Challenger Deep, the Mariana Trench, shown by colored bars.

In qPCR analyses, we found a gap between the relative abundances of total archaeal *amoA* and the archaeal SSU rRNA gene; total archaeal *amoA* was 27–45% of the archaeal SSU rRNA gene abundance above 65 cmbsf and decreased to 6% and 0.8% at 100 and 123 cmbsf, respectively (Fig. 3). This gap was accounted for by the results of SSU rRNA gene tag sequencing using a primer mixture of 505F/907R, but cannot be explained by the results of the classical SSU rRNA gene clone analysis, which revealed the predominance of the MGI thaumarchaeal population in all samples (Fig. 5, Table S3 and S5). In the tag analysis, the relative abundance of tags related to MGI thaumarchaeotes in archaeal SSU rRNA gene tags ranged between 54 and 75% in sediments above 65 cmbsf, but was only 2% in sediments from 123 cmbsf, and the predominance of the “*Ca.* Woesearchaeota” (DHVE6) population was observed in deeper sections. The possible underestimation of “*Ca.* Woesearchaeota” in the archaeal SSU rRNA gene communities using the archaea-specific primer set Arch21F/Arch958R was also revealed in a similar comparison for another subseafloor environment (43). Accordingly, the archaeal community structures dominated by “*Ca.* Woesearchaeota” shown by SSU rRNA gene tag sequencing were likely to be less biased than those shown by the SSU rRNA gene clone analysis.

### Nitrogen cycle in Challenger Deep sediments

Molecular analyses revealed that the maximum abundance of ammonia-oxidizing archaea (AOA) and nitrite-oxidizing bacteria (NOB) occurred in surface sediments (Fig. 3). The abundance peaks of ammonia oxidizers (AOA and ammonia-oxidizing bacteria [AOB]) and NOB were also observed at 45 cmbsf. The distribution pattern was consistent with the stable isotopic signatures of NO_3_^−^ that suggest the co-occurrence of nitrification and denitrification above 45 cmbsf (Fig. 2). In contrast, the peak of NO_2_^−^ at 65 cmbsf was likely to be the result of partial denitrification (Fig. 1). On the other hand, the correlation between the decreasing population of *Nitrospira* and increasing concentration of NO_2_^−^ suggests that the shallow NO_2−_ peak was the result of ammonia oxidation; however, it currently remains unclear why the *Nitrospira* population decreased at this depth (Fig. 1, 2, and 3).

The unsuccessful amplification of *hzsA* suggests that anammox is not a major player in the nitrogen cycle in the trench bottom sediment. Based on the presence of sufficient amounts of NO_2_^−^ at depths at which denitrification outcompeted aerobic nitrification, low NH_4_^+^ concentrations may be a limiting factor for the development of the anammox bacterial population in this sedimentary environment (Fig. 1 and 3).

### Comparison between hadal water and sediment microbial communities

The relationship between microbial communities in bottom sediments and the overlying deep-sea waters has been rarely investigated in hadal environments. Cell density was two orders of magnitude larger in surface sediments than in hadal water (41) (Fig. 3). Based on a comparison of SSU rRNA gene tag communities, MGI *Thamumarchaeota*, *Planctomycetes*, *Gemmatimonadetes*, “*Ca.* Marinimicrobia” (SAR406), and *Bacteroidetes* were commonly abundant in hadal water 50 m above the trench bottom and in the bottom sediment at the same sampling station (41) (Fig. 4). The relative abundances of MGI, SAR324, and *Chloroflexi* SAR202 in the SSU rRNA gene tag community were higher in hadal sediments than in trench bottom water, whereas these groups are known to be the representative lineages of oceanic waters (52). In contrast, “*Ca.* Pelagibacterales” (SAR11), one of the predominant lineages in oceanic waters, was absent in the benthic communities.

Distance matrices were created using the UniFrac program to compare the tag communities from trench bottom water and sediment samples (Fig. 5). The results obtained indicated that the tag communities, including the trench bottom water community, were similar to each other at higher taxonomic levels. Similarities among the tag communities were also tested by two dissimilarity indices: Bray-Curtis and Jaccard (Fig. 5). Overall, the Jaccard index for the SSU rRNA gene tags implied that tag populations in hadal water and sediments were distinct from each other; however, shared populations were found in some of the abundant taxa/divisions. Bray-Curtis dissimilarity based on the abundance of each OTU also showed similar results. These distance matrices were tested for each of the dominant taxa/divisions (Fig. S1, S2, and S3). As an exception, the benthic thaumarchaeal population was similar to that in trench bottom water at the species or genus level. On the other hand, the UniFrac index indicated that the abundant populations of *Acidobacteria*, *Chloroflexi*, and “*Ca.* Woesearchaeota”, in trench bottom water were similar to those in hadal sediments at a higher taxonomic level, whereas those of the other dominant taxa in hadal water were distinct from those in sediments.

Hadal water and sediment MGI populations were dominated by similar phylotypes (less than 2% dissimilarity) that belonged to the same species or genus (less than 3 and 5% dissimilarity, respectively) of group α MGI in the Challenger Deep, whereas diverse group α MGI phylotypes were detected in hadal sediments in the Ogasawara Trench (40). Among the MGI subgroups, niche separation appears to be primarily controlled by the availability of ammonia (41, 56). Groups δ and γ MGI, which adapt to low ammonia concentration/flux, predominated the microbial community in the overlying abyssal water, while group α became abundant in the hadal water ecosystem and appeared to be supported by suspended organic carbon from the trench slope (41, 42). In the case of the Mariana Trench, hadal environments are topographically isolated from other trench environments (19). In addition, hydrostatic pressure in the hadal environment may be a bottleneck for selecting specific populations. Thus, the immigration and adaptation of the extrinsic microbial population in hadal environments appear to be limited. As a result of highly biased selection, a single species or genus that adapts to hadal environments may dominate the microbial communities in water and sediment habitats in the Challenger Deep. Intensive microbiological investigations on the Mariana Trench, including this study, hypothesize that the trench geomorphological and geographic relationship among the hadal trenches may regulate not only the biogeochemical cycle in the hadal biosphere, but also the microbial community compositions and biogeographic diversification of microbial populations in the planktonic and benthic habitats of hadal trenches.

## Supplementary Material
