# Supplementary material for: Microbial Diversity in Sediments from the Bottom of the Challenger Deep, the Mariana Trench
Source: Microbes Environ. 2018 May 25;33(2):186–94. doi: 10.1264/jsme2.ME17194 (PMC6031389; doi:10.1264/jsme2.ME17194)
Supplement: Supplementary file 1 [file 33_186_s1.pdf]

Supplementary Information

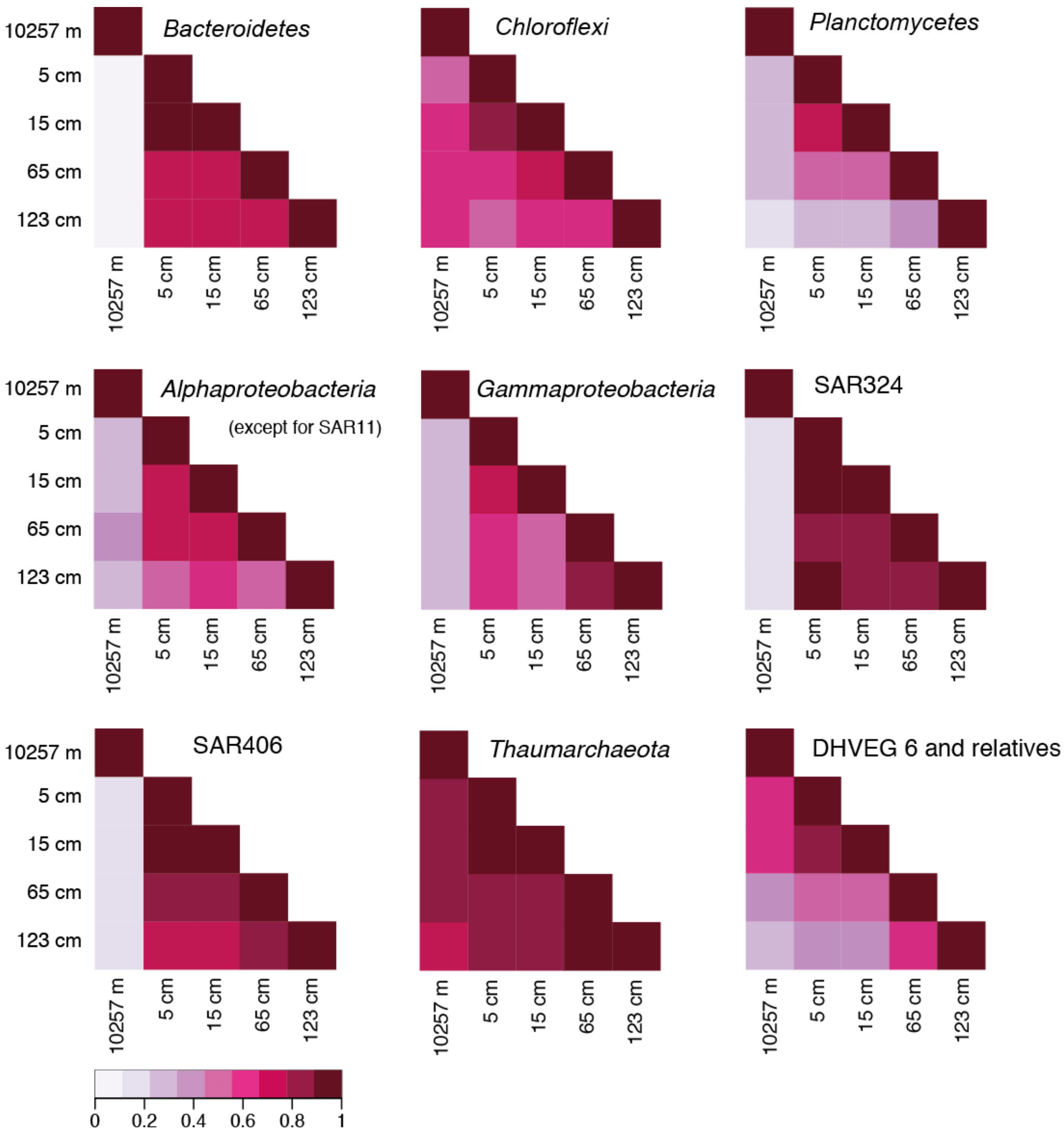

Fig. S1. UniFrac matrices of dominant taxa/divisions found in the SSU rRNA gene tag sequence analysis for the trench bottom sediments in the Challenger Deep, Mariana Trench.

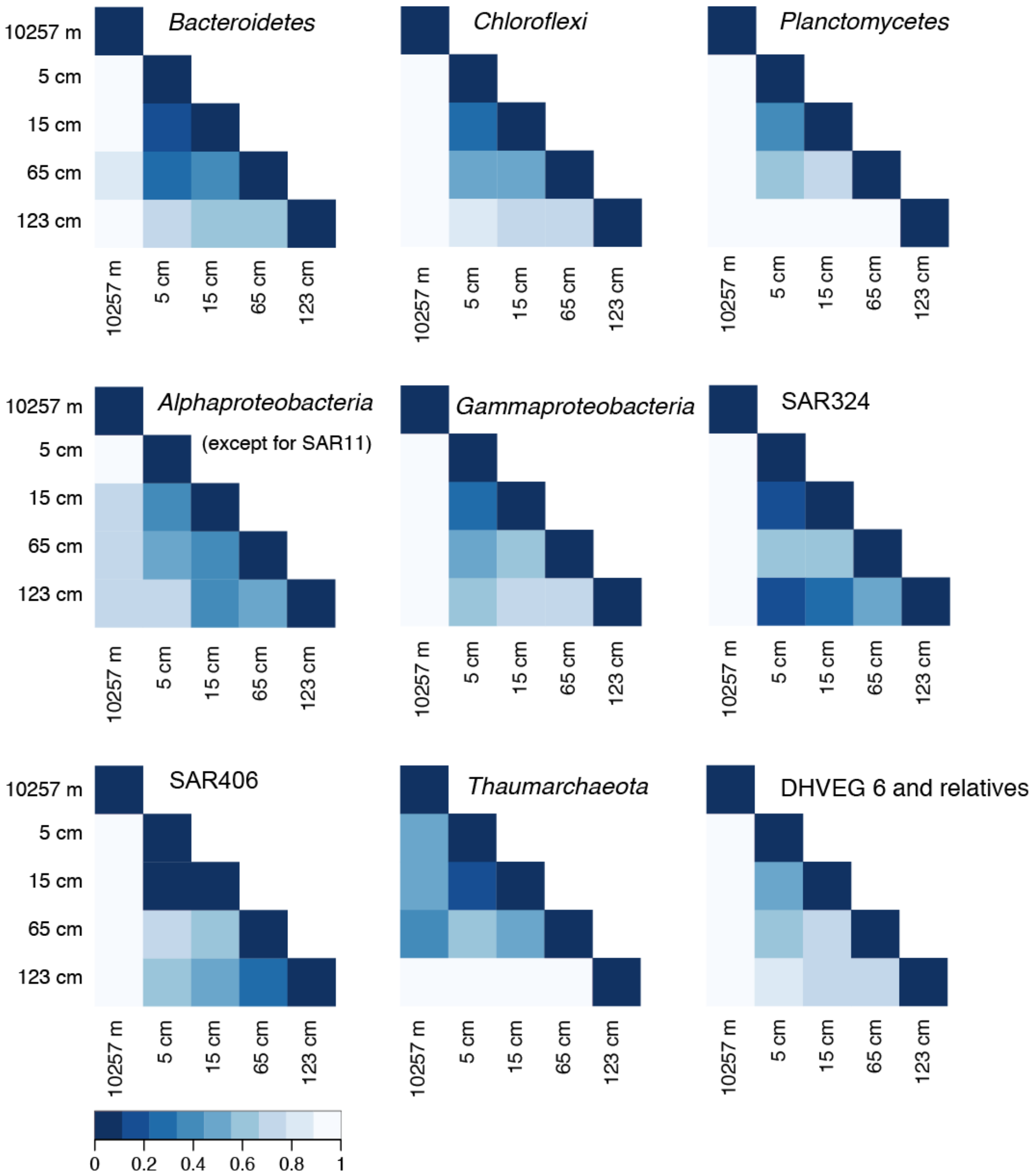

Fig. S2. Bray-Curtis dissimilarity matrices of dominant taxa/divisions found in the SSU rRNA gene tag sequence analysis for the trench bottom sediments in the Challenger Deep, Mariana Trench.

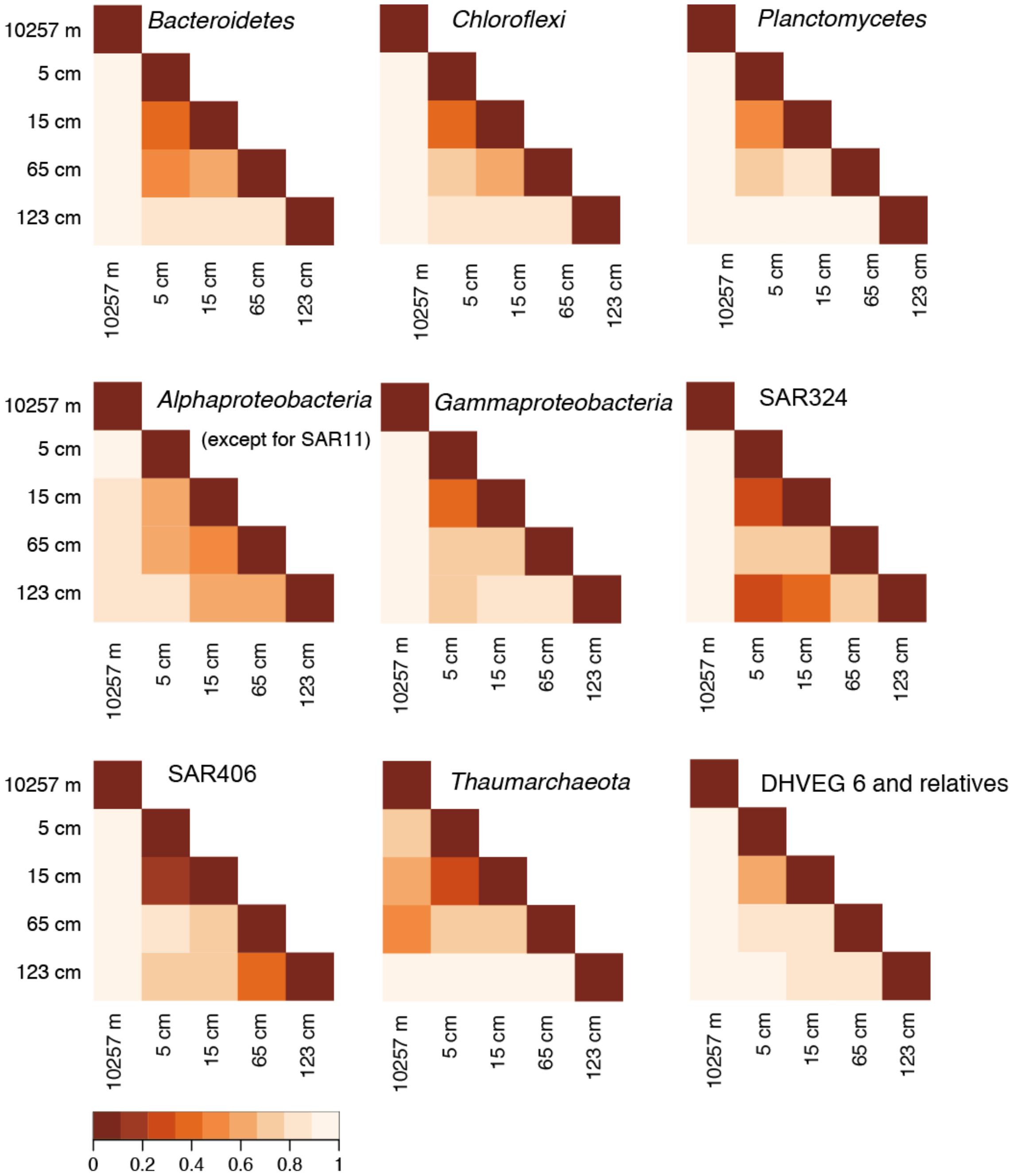

Fig. S3. Jaccard dissimilarity matrices of dominant taxa/divisions found in the SSU rRNA gene tag sequence analysis for the trench bottom sediments in the Challenger Deep, Mariana Trench.

Table S1. PCR conditions, and primer and probe sequences used in this study.

Target genes	Primers	Sequence (5' 3')	PCR conditions	Standard mixture for quantitative PCR	References
**Clone analysis and detection**					
Archaeal SSU rRNA gene	A21F	TTCCGGTTGATCCYGCCGGA	96˚C for 1 min, 30 x (96˚C 25s, 50˚C 45 s,72˚C 90 s)		DeLong, 1992
	A958R	YCCGGCGTTGAMTCCAATT			
Bacterial SSU rRNA gene	B27F	AGAGTTTGATCCTGGCTCAG	96˚C for 1 min, 25 x (96˚C 25 s, 53˚C 45 s,72˚C 90 s)		Lane, 1985
	B927R	CCGCTTGTGCGGGCCC			DeLong et al, 1989
Archaeal *amoA*	Arch-amoAF	STAATGGTCTGGCTTAGACG	96˚C for 5 min, 30 x (96˚C 25 s, 52˚C 30 s,72˚C 1 min)		Francis *et al*., 2005
	Arch-amoAR	GCGGCCATCCATCTGTATGT			
Anammox bacteria *hzsA*	hzsA_526F	TAYTTTGAAGGDGACTGG	96˚C for 1 min, 35 x (96˚C 30 s, 54˚C 60 s, 72˚C 90 s)		Harhangi *et al*., 2012
	hzsA_1857R	AAABGGYGAATCATARTGGC			
**Tag sequencing**					
SSU rRNA gene	530F	Mixtrures	96 ˚C for 1 min, and 25 to 40 cycles of 96˚C for 20 s, 48˚C for 70 s and 72˚C for 30 s		Nunoura et al. 2012
	907R	Mixtrures			
**Quantitative PCR**					
Prokaryotic SSU rRNA gene	Uni340F	CCTACGGGRBGCASCAG	96˚C for 1 min, 50 x (96˚C 25 s, 57˚C 6 min)	Standard mixture (Takai & Horikoshi 2000)	Takai and Horikoshi, 2000
	Uni516F	TGYCAGCMGCCGCGGTAAHACVNRS			
	Uni806R	GGACTACNNGGGTATCTAAT			
Archaeal SSU rRNA gene	Arch349F	GYGCASCAGKCGMGAAW	96˚C for 1 min, 50 x (96˚C 25 s, 59˚C 6 min)	Standard mixture (Takai & Horikoshi 2000)	Takai and Horikoshi, 2000
	Arch516F	TGYCAGCCGCCGCGGTAAHACCVGC			
	Arch806R	GGACTACVSGGGTATCTAAT			
Nitrospina-like SSU rRNA gene	Nspina16S_283F	TGKWTAGCCRGCCTGA	94˚C for 5 min,40 x (94˚C 15 s, 53˚C 15 s, 72˚C 34 s)	Standard mixture (Nunoura et al. 2013)	Nunoura et al. 2013
	Nspina16S_776R	CCCTRGCTTTCGTATCT			
Nitrospina related group (SFNLG) SSU rRNA gene	Nspina16S_283F	TGKWTAGCCRGCCTGA	94˚C for 5 min, 40 x (94˚C 15 s, 53˚C 15 s, 72˚C 34 s)	Standard mixture (Nunoura et al. 2013)	Nunoura et al. 2013
	Nspina_rela16S_847R	ATCGATACCTCCTTCACC			
Archaeal *amoA* group A	Group A 76F	ACTGAYTGGGCCYTGGACATCG	94˚C for 2min, 40 x (96˚C 20s, 58˚C 10s, 72˚C 34s)	Standard mixture (Nunoura et al. 2015)	Nunoura et al. 2015
	Group A 415R	AATGCAGTCTCDARYGGRTCWGCC			
Archaeal amoA group Ba	Group Ba 138F	GGTGCWGTGTATTATCTYACATTTAC	94˚C for 2min, 40 x (96˚C 20s, 58˚C 10s, 72˚C 34s)	Standard mixture (Nunoura et al. 2015)	modified from Beman et al. 2008
	Group Ba 538R	AAYGTACAYGCTAGTACMGMTCC			Nunoura et al. 2015
Archaeal amoA group Bb	Group B 274F	CAYCCDATGTGGATWCCATCDTG	94˚C for 2min, 40 x (96˚C 20s, 58˚C 10s, 72˚C 34s)	Standard mixture (Nunoura et al. 2015)	Nunoura et al. 2015
	Group Bb 546R	ARTGCYGCAAAYGTYACYGAYAG			
Archaeal *amoA* group D	Group D 191F	SYCTNATHATGACAGTATACAC	95˚C for 5min, 50 x (96˚C 20s, 56˚C 15s, 72˚C 34s)	Standard mixture (Nunoura et al. 2015)	Nunoura et al. 2015
	Group D 436R	TGGYCTYGGRTAYTTGAABGCC			
Betaproteobacterial *amoA*	*amoA*-1F	GGGGHTTYTACTGGTGGT	95˚C for 30 s, 40 x (95˚C 5 s, 54˚C 30 s, 64˚C 34 s)	Standard mixture (Nunoura et al. 2013)	Stephen *et al*., 1999
	amoA-2R	CCCCTCKGSAAAGCCTTCTTC			
Nitrospira SSU rRNA gene	Nspra675f	GCGGTGAAATGCGTAGAKATCG	95˚C for 10 min, 40 x (95˚C 20s , 58˚C 1 min, 72˚C 40 s)	Standard mixture (Nunoura et al. 2013)	Graham *et al*., 2007
	Nspra746r	TCAGCGTCAGRWAYGTTCCAGAG			
	Nspra-723Taq	CGCCGCCTTCGCCACCG			

Table S2. Pore water geochemistry of core #AB11 taken from the bottom of the Challenger Deep, Mariana Trench.

Sections (cmbf)	pH	Alkalinity (mM)	NO_3^- (μM)	NO_2^- (μM)	NH_4^+ (μM)	PO_4^{3-} (μM)	SO_4^{2-} (mM)	δ15N NO_3^-	δ18O NO_3^-
Bottom Water			34.84	0.60	0.58	1.055		4.4	3.9
0-5	7.69	1.75	38.56	0.41	2.71	0.888	27.93		
5-10	7.73	2	38.05	0.32	2.83	2.784	28.20	6.6	2.8
10-15	7.76	2.25	36.76	3.44	6.55	3.702	27.65		
15-20	7.79	2.25	35.44	1.25	2.50	3.789	27.76	7.9	6.2
20-30	7.8	2.25	29.89	0.14	0.34	4.086	27.81	7.6	7.3
30-40	7.85	2.25	27.46	0.17	1.30	4.314	27.74	8.2	8.4
40-50	7.84	2.5	25.72	0.68	2.65	5.169	27.58	8.9	10.1
50-60	7.91	2.5	18.79	2.72	2.05	5.262	27.59	14.1	15.6
60-70	7.91	2.5	13.51	3.20	1.54	4.950	27.57	18.0	18.1
70-80	7.99	2.75	9.31	2.99	1.48	5.796	27.46	19.2	20.5
80-90	7.98	2.75	4.99	2.09	2.59	6.456	27.40	17.8	20.7
90-104	7.94	3	5.26	1.25	2.47	5.598	27.25		
104-113	7.99	2.75	3.34	0.53	1.96	1.821	27.43		
113-120	7.99	3	4.00	0.35	3.67	1.992	27.24		
120-127	7.97	3	3.67	0.11	5.83	1.635	27.36		

Table S3. Distribution of the representative archaeal SSU rRNA gene sequences obtained from the trench bottom sediment from the Challenger Deep, Mariana Trench.

Phylotypes	Depth (cmbsf)					
	5 cm	12.5 cm	45 cm	65 cm	100 cm	123 cm
Thaumarchaeota						
MGI						
MCD_AB10_5cm_A01	46	44	48	47	47	85
MCD_AB10_123cm_A11						1
FSCG						
MCD_AB10_123cm_A27						1
Euryarchaeota						
Lost City *Methanosarcinales*						
MCD_AB10_123cm_A10						3
Total	46	44	48	47	47	90

Table S4. Distribution of the representative bacterial SSU rRNA gene sequences obtained from the trench bottom sediment from the Challenger Deep, Mariana Trench.

Phylotypes	Depth (cmbsf)					
	5 cm	12.5 cm	45 cm	65 cm	100 cm	123 cm
Alphaproteobacteria						
MCD_AB10_5cm_B30	3		2	1	1	
MCD_AB10_5cm_B37	1	2				
MCD_AB10_12.5cm_B06		3			2	3
MCD_AB10_12.5cm_B22		1	2	2	2	2
MCD_AB10_12.5cm_B35		1				1
MCD_AB10_45cm_B11			1			
MCD_AB10_45cm_B24			1	1		
MCD_AB10_45cm_B43			1	2		
MCD_AB10_45cm_B45			1			
MCD_AB10_45cm_B46			1			
MCD_AB10_65cm_B45				1		
MCD_AB10_100cm_B11					2	1
MCD_AB10_123cm_B42						2
Gammaproteobacteria						
MCD_AB10_5cm_B01	1					
MCD_AB10_5cm_B02	1		1			
MCD_AB10_5cm_B25	1	1	1	1		
MCD_AB10_12.5cm_B39		1				
MCD_AB10_45cm_B31			1			
MCD_AB10_45cm_B33			1			
MCD_AB10_45cm_B35			1			
MCD_AB10_45cm_B36			1			
MCD_AB10_65cm_B11				1		
MCD_AB10_65cm_B28				1		
Deltaproteobacteria						
MCD_AB10_12.5cm_B10		1		2		
MCD_AB10_12.5cm_B28		1		1		
MCD_AB10_45cm_B20			1			
MCD_AB10_65cm_B22				1		
MCD_AB10_65cm_B36				1		
MCD_AB10_65cm_B39				1		
MCD_AB10_123cm_B46						1
SAR324						
MCD_AB10_5cm_B45	1	1			1	1
MCD_AB10_12.5cm_B30		1				1
other *Proteobacteria*						
MCD_AB10_5cm_B03	2	1	2	1	1	3
Acidobacteria						
MCD_AB10_5cm_B38	1			4		
MCD_AB100cm_B03					1	
Actinobacteria						
MCD_AB10_65cm_B15				2	4	2
MCD_AB10_65cm_B31				1		
Bacteroidetes						
MCD_AB10_5cm_B33	1		1			
MCD_AB10_12.5cm_B48		1				
MCD_AB10_45cm_B47			1		1	1
MCD_AB10_65cm_B38				1		
MCD_AB10_65cm_B47				1		
MCD_AB10_123cm_B03						1
Chloroflexi						
SAR202 cluster						
MCD_AB10_5cm_B4	2					
MCD_AB10_5cm_B17	1	3	1	1	2	
MCD_AB10_5cm_B24	1					
MCD_AB10_5cm_B32	1	1	2			
MCD_AB10_12.5cm_B09		1	1	3	1	
MCD_AB10_12.5cm_B25		1				
MCD_AB10_12.5cm_B37		2				
MCD_AB10_12.5cm_B38		1				
MCD_AB10_12.5cm_B40		1				
MCD_AB10_45cm_B21			1	1		
MCD_AB10_45cm_B23			1			
MCD_AB10_45cm_B37			1			
MCD_AB10_45cm_B42			1		1	1
MCD_AB10_100cm_B35					1	

MCD_AB10_123cm_B21						1
other *Chloroflexi*						
MCD_AB10_5cm_B07	5	2		1	2	1
MCD_AB10_5cm_B12	2		1			
MCD_AB10_5cm_B21	1		1	3	2	
MCD_AB10_5cm_B34	1					
MCD_AB10_12.5cm_B04		1				
MCD_AB100cm_B08					1	
MCD_AB100cm_B10					2	
MCD_AB10_100cm_B25					1	
MCD_AB10_123cm_B08						2
MCD_AB10_123cm_B16						1
MCD_AB10_123cm_B17						1
MCD_AB10_123cm_B19						3
MCD_AB10_123cm_B29						1
MCD_AB10_123cm_B33						1
MCD_AB10_123cm_B35						1
MCD_AB10_123cm_B48						1
Gemmatimonadetes						
MCD_AB10_5cm_B09	3	2	2	3	4	1
MCD_AB10_5cm_B11	3	1	6	5		
MCD_AB10_123cm_B25						2
MCD_AB10_123cm_B37						1
other bateria						
MCD_AB10_5cm_B05	1					
MCD_AB10_5cm_B08	1					
MCD_AB10_5cm_B10	1					
MCD_AB10_5cm_B14	1					
MCD_AB10_5cm_B18	1	2			1	
MCD_AB10_5cm_B23	1					
MCD_AB10_5cm_B27	1					
MCD_AB10_5cm_B29	1					
MCD_AB10_5cm_B36	1					
MCD_AB10_5cm_B43	1					
MCD_AB10_5cm_B46	3	2				
MCD_AB10_5cm_B47	1					
MCD_AB10_12.5cm_B03		1			2	
MCD_AB10_12.5cm_B08		1				
MCD_AB10_12.5cm_B14		2				
MCD_AB10_12.5cm_B18		1				
MCD_AB10_12.5cm_B20		1				
MCD_AB10_12.5cm_B26		1				
MCD_AB10_12.5cm_B32		1				
MCD_AB10_12.5cm_B33		1				
MCD_AB10_12.5cm_B41		1				
MCD_AB10_12.5cm_B42		1				1
MCD_AB10_45cm_B13			1			
MCD_AB10_45cm_B17			1		1	
MCD_AB10_45cm_B32			1			
MCD_AB10_45cm_B44			1			
MCD_AB10_65cm_B17				1		
MCD_AB10_65cm_B19				1		
MCD_AB10_65cm_B30				1		
MCD_AB10_65cm_B42				1		
MCD_AB10_100cm_B33					1	
MCD_AB10_100cm_B42					4	
MCD_AB10_100cm_B46					1	
MCD_AB10_123cm_B07						1
MCD_AB10_123cm_B09						1
MCD_AB10_123cm_B11						3
MCD_AB10_123cm_B43						1
Total	46	46	42	47	42	44

Table S5. Summary of SSU rRNA gene tag sequencing of the hadal water and trench bottom sediment in the Challenger Deep, Mariana Trench.

		Water depth (m)	Sedeiment depth (cmbsf)			
		10257	5	15	65	123
Bacteria						
Alphaproteobacteria	SAR11	48	0	0	0	0
	Sphingomonadales	5	3	2	7	2
	Rhizobiales	4	42	93	61	7
	Rhodobacterales	120	20	19	42	3
	Rhodospirillales	430	279	444	561	609
	others	18	55	55	44	26
Betaproteobacteria	*Nitrosomonas*	9	3	2	10	0
	others	11	1	0	4	0
Deltaproteobacteria	SAR324 (*Bdellovibrionales*)	14	252	195	103	204
	other *Bdellovibrionales*	14	2	11	1	1
	Desulfobacterales	8	44	78	30	97
	Myxococcales	8	24	18	17	1
	others	96	46	52	215	9
Gammaproteobacteria	*Alteromonadales*	20	3	0	5	2
	Oceanospirillales	7689	96	146	80	17
	Pseudomonadales	2106	0	4	0	4
	Xanthomonadales	16	118	112	220	61
	Bd7-8_marine_group	0	7	2	85	7
	others	13	36	22	146	36
Zetaproteobacteria		1	50	31	24	1
other *Proteobacteria*	JTB23	8	478	388	405	349
	others	28	16	6	12	0
Acidobacteria		70	41	23	68	135
Actinobacteria		10	96	78	137	319
Armatimonadetes		0	47	58	8	25
Bacteroidetes		1039	822	632	1334	311
Chlorobi		0	141	212	283	2
Chloroflexi	SAR202	59	790	929	461	688
	others	6	1096	749	922	3595
Deferribacteretes		30	32	33	43	370
Elusimicrobia		0	14	28	16	65
Firmicutes		1	27	39	153	264
Gemmatimonadetes		148	448	352	428	361
"Ca. Marinimicrobia" (SAR406)		1299	487	572	494	782
Nitropinae	*Nitrospina*	2	29	49	25	10
Nitrospirae	*Nitrospira*	32	6	10	5	1
	others	0	1	2	3	34
Spirochaete		0	5	3	1	15
" *Ca*. Parcubacteria" (OD1)		1	47	120	23	83
Planctomycetes		717	1181	990	907	757
Verrucomicrobia		4	14	13	6	1
BD1-5		0	36	40	16	24
Bhi80-139		0	0	0	0	684
BRC1		0	91	60	80	14
OP3		0	48	27	31	31
TA06		0	68	189	34	42
TM6		0	21	14	18	4
WS3		0	1	0	2	77
other *Bacteria*		6	48	41	20	57
Archaea						
Euryarchaeota						
Methanomicrobia		0	11	2	0	0
Thermoplasmata		11	4	13	1	3
DSEG		0	4	38	1	1
other *Euryarchaeota*		0	3	0	0	0
"*Ca*. Woesearchaeota" (DHVEG 6)		42	1199	2197	1110	2162
Thaumarchaeota	*Nitrosopumilales* (MGI)	1536	3622	2646	1828	47
	Marine Benthic Group A (FSCG)	0	14	23	12	2
	other *Thaumarchaeota*	0	0	0	1	0
Other *Archaea*		0	1	0	0	0
Others		0	4	1	4	0
Total		15679	12074	11863	10547	12402

Table S6. Distribution of the representative archaeal *amoA* gene sequences obtained from the trench bottom sediment from the Challenger Deep, Mariana Trench.

Phylotypes	Depth (cmbsf)					
	5 cm	12.5 cm	45 cm	65 cm	100 cm	123 cm
A						
MCD_AB10_AmoA_65cm_19	3	1	4	2	4	3
MCD_AB10_AmoA_100cm_06	1	1		2	2	2
MCD_AB10_AmoA_100cm_27		1			2	
MCD_AB10_AmoA_100cm_29			1		1	
MCD_AB10_AmoA_123cm_29						1
Ba						
MCD_AB10_AmoA_45cm_13	2	1	1			
MCD_AB10_AmoA_100cm_12			1		2	
MCD_AB10_AmoA_123cm_04						1
MCD_AB10_AmoA_123cm_10	17	26	14	26	19	24
Bb						
MCD_AB10_AmoA_5cm_11	1					
MCD_AB10_AmoA_123cm_25	1		2			1
C						
MCD_AB10_AmoA_100cm_14	1				2	
D						
MCD_AB10_AmoA_5cm_16	5	1	1			
MCD_AB10_AmoA_45cm_15			1			
MCD_AB10_AmoA_65cm_01			6	1		
MCD_AB10_AmoA_65cm_04				1		
Total	31	31	31	32	32	32
